# Preliminary investigation of plasma levels of sex hormones and human growth factor(s), and P300 latency as correlates to cognitive decline as a function of gender

**DOI:** 10.1186/1756-0500-2-126

**Published:** 2009-07-07

**Authors:** Eric R Braverman, Thomas JH Chen, Amanda LC Chen, Mallory M Kerner, Howard Tung, Roger L Waite, John Schoolfield, Kenneth Blum

**Affiliations:** 1 Path Medical Foundation, New York, NY, USA; 2 Department of Neurosurgery, Weill Cornell Medical College, New York, NY, USA; 3 Chang Jung Christian University, Tainan, Taiwan, Republic of China; 4 Changhua Christian Hospital, Changhua, Taiwan, PR China; 5 Department of Academic Informatics Services, University of Texas Health Science Center, San Antonio, Texas, USA; 6 LifeGen Inc, San Diego, CA, USA; 7 Department of Neurosurgery, Scripps Memorial Hospital, La Jolla, CA, USA; 8 Department of Physiology & Pharmacology, Wake Forest University School of Medicine, Winston-Salem, North Carolina, USA

## Abstract

**Background:**

Aging is marked by declines in levels of many sex hormones and growth factors, as well as in cognitive function. The P300 event-related potential has been established as a predictor of cognitive decline. We decided to determine if this measure, as well as 2 standard tests of memory and attention, may be correlated with serum levels of sex hormones and growth factors, and if there are any generalizations that could be made based on these parameters and the aging process.

**Findings:**

In this large clinically based preliminary study several sex-stratified associations between hormone levels and cognition were observed, including (1) for males aged 30 to 49, both IGF-1 and IGFBP-3 significantly associated negatively with prolonged P300 latency; (2) for males aged 30 to 49, the spearman correlation between prolonged P300 latency and low free testosterone was significant; (3) for males aged 60 to 69, there was a significant negative correlation between P300 latency and DHEA levels; (4) for females aged 50 to 59 IGFBP-3 significantly associated negatively with prolonged P300 latency; (5) for females at all age periods, estrogen and progesterone were uncorrelated with P300 latency; and (6) for females aged 40 to 69, there was significant negative correlation between DHEA levels and P300 latency. Moreover there were no statistically significant correlations between any hormone and Wechsler Memory Scale-III (WMS-111). However, in females, there was a significant positive correlation between estrogen levels and the number of Attention Deficit Disorder (ADD) complaints.

**Conclusion:**

Given certain caveats including confounding factors involving psychiatric and other chronic diseases as well as medications, the results may still have important value. If these results could be confirmed in a more rigorously controlled investigation, it may have important value in the diagnosis, prevention and treatment of cognitive impairments and decline.

## Introduction

A review of the literature reveals that sex hormones have often been associated with changes in behavioral and mental abilities. As noted by Craig and Murphy, estrogen may modulate brain function, and acute loss of ovarian hormones increases neuronal membrane breakdown. Additionally, suppression of ovarian function may reduce activation of brain regions that are critical for memory [1]. Other research has shown that estrogen use may improve mood amongst women with postnatal or perimenstrual depression; however, it may contribute to increasing depressive symptoms in women with premenstrual dysphoria [2]. Although it has yet to become widely accepted, research has shown that estrogen replacement therapy may help prevent Alzheimer's dementia [3].

The behavioral effects of the androgens testosterone and dehydroepiandrosterone (DHEA) remain unclear but preliminary reports suggest that their use is associated with improved mood [4,5]. There is evidence that there is an age-related testosterone depletion associated with the development of Alzheimer disease [6]. At present, there is not enough hard data to support the use of sex hormones and DHEA, the adrenal androgen, for the treatment of depression, cognitive decline or memory deficits.

The GH/insulin-like growth factor-1 (GH/IGF-1) axis is known to be involved in aging of physiological functions including low levels associating with cognitive decline as a function of age [7]. Deficiency of growth hormone (GH), an important regulator of IGF-1, is associated with reduced wellbeing [8]. Furthermore, since plasma IGF-1 levels have been reported to be enhanced by DHEA administration, it has been suggested that IGF-1 may have a role in some of the reported associations between low DHEA sulfate levels and impaired health measures in elderly subjects [9].

Since there is sparse definitive information on hormone plasma levels for measurement of cognitive decline as an age-related process, we decided to investigate this issue.

## Methods

Plasma sex and growth hormones (testosterone, DHEA, estrogen, progesterone, GH and IGF-1) were analyzed in both males and females across a wide age range and the results were statistically correlated with an electroencephalograph-obtained event-related potential (P300), whose prolonged latency has been established as an accurate predictor of cognitive and memory decline. The methods employed in this study were previously reported by our laboratory [10]. Subjects also took the Wechsler Memory Scale (WMSIII) and the Test of Variables of Attention (T.O.V.A.), which have been described in our previous work [10].

### Subjects

In this large (721 females and 654 males with an age range of 30–93 years) clinically based study, all patients were tested for their response to the P300 event-related potential (ERP) and screened for circulating hormones (testosterone, progesterone, estrogen, dehydroepiandrosterone (DHEA), and growth hormones IGF-1 and IGFBP-3). Mean ± standard deviation of age for females was 57.4 ± 15.1 and 55.8 ± 14.5 for males.

### Criteria for study inclusion

The patients had at least one P300 test. All test interpreters were blinded to other patient results. These patients were selected for study from an outpatient private medical clinical practice (Medical and Neuropsychiatric) and research foundation in New York City. All subjects signed an approved IRB consent form based on an approval from the PATH Medical IRB committee (registration # IRB00002334). Subjects were made aware that their results could be used in medical research and by signing the informed consent, they volunteered to participate in this study.

### Plasma Analysis

Venipuncture was done in nonfasting subjects between 8:30 AM and 7:30 PM at baseline examination. Assays were performed blinded to information on the subject. Plasma levels of estradiol and sex hormone-binding globulin were estimated with double anti-body radioimmunoassays (Bioreference Lab, New York, New York). As measures of the levels of bioavailable and free estradiol, testosterone, DHEA, and nonprotein-bound estradiol, respectively, were calculated on the basis of hormone and binding protein levels. For the analysis of growth hormone and insulin-like growth factor binding proteins the laboratory performed standardized procedures. Free testosterone, or unbound testosterone, was calculated by correcting for SHBG and albumin.

### BEAM Mapping

All patients in this study were analyzed by the Brain Electrical Activity Map (BEAM), a computerized quantitative EEG. A 24-channel EEG recorder was used. Standard techniques were used to measure event-related P300 prolonged latency [10]. The standard International 10/20 System of electrode placement was used. In addition, there were two on earlobes, two EKG electrodes connected to the cervical spine and electrocap, and two supraorbital electrodes (EOG) were also used. Digital EEG was recorded in a monopolar (LR linked ears left over right) and bipolar (LR 3,4 linked ears left over right) montage. Waveforms were averaged off-line, such that trials on which the EEG or EOG exceeded ± 100 microvolts were rejected; single-trial data also were subjected to an EOG correction procedure to remove any remaining artifact.

### Statistics

As hormone levels are known to be influenced by sex, the analysis was performed for females and males separately. For females, IGF-1, IGFBP-3, estrogen, progesterone, and DHEA were studied. For males, IGF-1, IGFBP-3, free testosterone, and DHEA were studied. Since the study included a broad spectrum of ages, the potential for the age factor to confound the analysis of the complete data set compelled an approach in which subjects were stratified by age decade (i.e. 30 to 39, 40 to 49, etc.). This approach helped to negate any influence that age might have on P300 latency and hormone levels, since it has been previously established that the distribution of these measures are not independent of age [10]. Also, levels of certain hormones may be important diagnostically for specific age groups, while, for other age groups, hormone variability may be suppressed by the age factor. To ensure sufficient sample size for each strata, the age groups selected were 30 to 39 years, 40 to 49 years, 50 to 59 years, 60 to 69 years, and 70 and above, with a maximum age of 93 years. Within each age group, the sample lower quartile (25^th ^percentile) was obtained for each hormone level and used as a cutpoint to define low hormone levels. The hormone data was standardized by assigning a percentile ranking of 1 to 20 to each hormone measure, with 1 representing values ranging from the minimum to the 5^th ^percentile and 20 representing values ranging from the 95^th ^percentile to the maximum. Partial correlations were performed to check for associations between hormone rankings and P300 latency controlling for age. As a consequence of previous analyses, the subject's age plus 300 served as the cutpoint defining prolonged P300 latency for all subjects [10]. Using the cutpoints, contingency table analysis was performed to determine if a difference in the percentage of subjects with low hormone levels was observed for prolonged vs. normal P300 latency, with p < 0.05 for the Chi-square test considered significant. To further substantiate possible associations between P300 latency and hormone levels within an age group, Spearman correlation was performed. If adjacent age groups had similar results the age groups were combined to obtain generalized results.

## Results

While the amplitude of the P300 evoked potential was assessed in this study we did not find any significant associations. However, in terms of latency, significant associations included (1) in males aged 30 to 49, both IGF-1 (rho = -0.357, p < 0.001) and IGFBP-3 (rho = -0.230, p == 0.034) significantly associated negatively with prolonged P300, and for all males, rank-transformed IGF-1 and IGFBP-3 values were significantly correlated (r = 0.521, p < 0.001) after partially out the age effect; (2) in males aged 30 to 49, the correlation between prolonged P300 latency and low free testosterone (rho = -0.305, p = 0.004) was significant; (3) in males aged 60 to 69, there was a significant negative correlation (rho = -0.345, p = 0.031) between P300 latency and DHEA levels; (4) in females aged 50 to 59, IGFBP-3 was significantly (rho = -0.239, p = 0.040) negatively associated with prolonged P300 latency; (5) In females aged 40 to 69, there was nearly significant **(rho = -0.170, p = 0.050) **negative correlation between DHEA levels and P300 latency. Moreover, for all females, there was a significant (rho = 0.352, p < 0.001) positive correlation between estrogen levels and the number of ADD complaints.

## Discussion

As stated this is a preliminary investigation and future studies will attempt to provide additional required information to make any serious interpretation of the current data. There are many caveats to this study that will provide a blueprint for additional meaningful research. One major problem in this study was the patient group being not well defined due to constraints of the clinical data collection center. While we are cognizant that a perspective study utilizing only healthy volunteers is warranted, the present study does have important value. With that stated our concern is that underlying disease is a major confounding factor because of the presence of a psychiatric disorder (as was the case for the majority of patients in this study). Other clinical disorders of note, such as Alzheimer's disease, diabetes mellitus, and chronic hepatic disease, among others, will affect the P300 measurements. Another caveat is that it is well known that several medications (for instance oral contraceptives in females) and also underlying diseases will influence measured hormone levels. Thus in future studies we will attempt to separate patients with chronic diseases and assign categories based on psychiatric disorders, and metabolic diseases where baseline values may be outliers, or further confounded by medication. In addition recent data suggest that head trauma is an important cause of pituitary dysfunction and also a well-known reason for cognitive dysfunction. In future studies the head trauma patients will be excluded from the study group, or at least those that fall into a <25^th ^percentile. We are also cognizant of the relationship of body mass index (BMI) of the patients on hormone levels. In that regard we have already collected data on over 900 patients and evaluated the relationship between growth factors and BMI as well as growth hormone secretion. In future studies we intend to exclude all patients with a BMI of >30. Moreover we are cognizant that many studies only measure hormone levels in the morning to avoid variation as a consequence of diurnal rhythms. This potentially confounding factor must be taken into account as another caveat of the present study. Importantly this fact is particularly true for testosterone.

Considering these caveats, our results can be analyzed with caution. In terms of the findings related to the female sex hormone estrogen and the P300 latency results, the literature has not consistently shown that estrogens prevent cognitive decline or Alzheimer's disease. In fact one study showed a small negative effect of higher estradiol levels in memory performance in both females and males and other studies do not support a positive outcome with hormone therapy [11]. In terms of the present study it is noteworthy, that binding sites for GH and IGF-1 contribute to the function of various brain areas. Their distribution suggests that GH and IGF-1 contribute to the function of the hippocampus, a brain structure important for the maintenance of cognitive functions such as learning and memory [12]. Evidence for cognitive deficits in GH-deficient individuals has been found in various studies, some of which have shown that these deficits can be reversed by GH substitution therapy [13]. Based on available data, one might hypothesize that relative GH or IGF-1 deficiency could contribute to the deterioration of cognitive function observed in the elderly. However, we cannot as yet provide an explanation for the differential gender findings with regard to the fact that IGFBP-3 significantly associated with P300 latency in females but IGF-1 did not, whereas both growth hormones significantly associated with P300 prolonged latency in males. Evidently it appears that both DHEA and free testosterone level negatively correlate with prolonged P300 latency especially in older men. Similar findings were obtained for DHEA levels and prolonged P300 latency in older women. These findings could have very significant importance in targeting both prevention and treatment of cognitive dysfunction in both males and females.

The present study also addressed attentional complaints and the potential relation to hormone levels. Follow up to this pilot study on attentional complaints further confirms the early findings. Attention problems have a multimodal dimension. ADHD, including attentional complaints, is related to memory, electrophysiology, and also to genetic and psychiatric factors. Attentional complaints also have Advanced Psychiatric Disease Performance Test (Axis I) sources, such as anxiety disorders, depression, schizophrenia, delusions, etc. It turns out that attention deficits and impaired memory are common to patients with depression [14]. The only hormone we found in the present pilot study to significantly associate with ADD complaints was estrogen. But it is also noteworthy that Lijffijt et al found that a reduction in attention-related brain potential as measured on an EEG, as well as marked selective attention impairments, were present in patients with reduced levels of GH and IGF-1 [15].

## Conclusion

The present study does not reject the possibility that exogenous estrogens, testosterone, DHEA, and growth hormones may be beneficial in protecting against cognitive decline. To our knowledge this is the first report showing a significant negative association of IGFBP-3, free testosterone and DHEA, with P300 prolonged latency (a predictor of memory impairment and cognitive decline) [10]. In addition, it is also the first report of a significant negative association of endogenous levels of estrogen and the number of ADD complaints in a clinical setting. In support of these results two recent studies clearly demonstrate that GH deficiency and low IGF-1 levels are associated with prolonged P300 latency in both females and males [16,17]. If these preliminary results could be confirmed, it may have important value in the diagnosis, prevention and treatment of early cognitive dysfunction.

## Competing interests

The authors declare that they have no competing interests.

## Authors' contributions

ERB developed the original concept and contributed to the development of the data set. TJHC provided input into the clinical protocol and contributed to the development of the manuscript. AC contributed equally to TJHC and provided additional literature references. RLW was involved in editorial assistance as well contributing to the literature. HT was involved in the drafting of the manuscript. making editorial comments. MK was involved in acquisition of funding, collection of data, or general supervision of the research group, JS participated in the design of the study and performed the statistical analysis. KB was involved in the design of the study, drafted the manuscript and was also involved in developing appropriate statistics as well as total overseeing of the project.
